# EROD and MROD as Markers of Cytochrome P450 1A Activities in Hepatic Microsomes from Entire and Castrated Male Pigs

**DOI:** 10.3390/s90302134

**Published:** 2009-03-23

**Authors:** Galia Zamaratskaia, Vladimir Zlabek

**Affiliations:** 1 Department of Food Science, Swedish University of Agricultural Sciences, SE-750 07, Uppsala, Sweden; E-mail: Vladimir.Zlabek@lmv.slu.se (V.Z.); 2 University of South Bohemia, Ceske Budejovice, Research Institute of Fish Culture and Hydrobiology, Zatisi 728, 389 25 Vodňany, Czech Republic

**Keywords:** Entire male pigs, castrated male pigs, porcine hepatic microsomes, EROD, MROD, HPLC, α-naphthoflavone, ellipticine, furafylline

## Abstract

In the present study, we characterized the kinetic parameters of 7-ethoxy-resorufin *O*-deethylation (EROD) and 7-methoxyresorufin *O*-demethylation (MROD) in hepatic microsomes from entire and castrated male pigs. Validation parameters of an HPLC-based method to analyse EROD and MROD activities are also described. Eadie-Hofstee plot analysis demonstrated a biphasic kinetic of EROD, indicating that at least two forms of cytochrome P450 are involved in this reaction. MROD followed monophasic kinetic, suggesting that a single enzyme, or enzymes with similar affinities, is responsible for the reaction. Inhibitory effects of α-naphthoflavone (ANF), ellipticine and furafylline were studied using microsomes from entire and castrated male pigs. ANF is a known inhibitor of both cytochrome P_450_ 1A1 and 1A2 (CYP1A1 and CYP1A2); the presence of ANF in the incubations resulted in the inhibition of both EROD and MROD activities in porcine liver microsomes. EROD activities in porcine liver microsomes were also inhibited by selective CYP1A1 inhibitor ellipticine, but not by CYP1A2 inhibitor furafylline. MROD activities were strongly inhibited by ellipticine and to a much lesser extent by furafylline. Further studies are needed to evaluate substrate specificities of porcine CYP1A1 and CYP1A2.

## 1. Introduction

Cytochrome P_450_ 1A (CYP1A) subfamily is commonly expressed in most animals and is of interest due to its ability to metabolically activate and inactivate some chemical carcinogens and environmental contaminants. In fish, CYP1A activities are often used as a marker to determine the quantities of persistent organic pollutants [1, 2].

Interest in investigations of cytochrome P_450_ (CYP) in porcine liver is growing because of the many similarities between porcine and human liver drug metabolizing enzymes [3] and thus the possibility to use the pig as an animal model in biomedical research. The CYP1A subfamily in mammals consists of two isoforms, CYP1A1 and CYP1A2, with more than 70% identity of amino acid sequences.

Enzymes CYP1A1 and CYP1A2 in pigs have received considerable attention in recent years. The full-length cDNA sequence encoding porcine CYP1A1 was determined and shown to have 85.4% similarity with human CYP1A1 [4]. Hepatic CYP1A1 and CYP1A2 in male and female Meishan pigs were investigated at levels of the mRNA, protein and enzyme activity [5]. A gender-related difference in the expression of hepatic CYP1A enzymes in Meishan pigs was demonstrated. Recently, enzymatic properties of porcine CYP1A2 were studied in the microsomes from β-naphthoflavone-treated male pigs [6].

Usually, the activities of CYP1A1 and CYP1A2 in different species are measured as a rate of the *O*-dealkylation of 7-ethoxy- and 7-methoxyresorufin for EROD and MROD, respectively [5, 7, 8]. The product of those reactions resorufin can be detected using the fluorimetric assay [8, 9] or high-performance liquid chromatography (HPLC) [10–12]. Recently, HPLC-based assays to measure EROD and MROD activities in liver microsomes from human, monkey, rat and mouse [10], and EROD activities in bovine liver microsomes [12] were fully validated.

Even though CYP1A1 and CYP1A2 are distinct, substrate specificities can overlap due to similarities between the active sites of CYP1A1 and CYP1A2 [13]. Additionally, the extrapolation of substrate specificities from one species to another is not always appropriate. It is therefore important to investigate substrate specificity for those enzymes in different species. The aim of the present study was to provide validation criteria for the analysis of EROD and MROD activities in porcine liver microsomes and to investigate kinetics of resorufin formation from 7-ethoxyresorufin and 7-methoxy-resorufin in hepatic microsomes from entire and castrated male pigs. The choice of pigs, entire *vs* surgically castrated, was based on the fact that surgical castration can modify activities of some cytochrome P_450_ enzymes [5, 14]. Additionally, we investigated *in vitro* inhibitory effect of α-naphthoflavone (ANF), ellipticine and furafylline on EROD and MROD activities.

## 2. Experimental Section

### 2.1. Chemicals, reagents and standard solutions

Resorufin, 7-ethoxyresorufin, 7-methoxyresorufin, α-naphthoflavone (ANF), ellipticine, furafylline, reduced β-nicotinamide adenine dinucleotide phosphate (NADPH) were obtained from Sigma-Aldrich (Steinheim, Germany). HPLC grade acetonitrile and methanol were purchased from Merck (Darmstadt, Germany). Stock solution of resorufin (4 mM) was prepared in methanol; stock solutions of 7-ethoxy-resorufin, 7-methoxyresorufin, ANF, ellipticine and furafylline were prepared in dimethylsulfoxide (DMSO). Aliquots of those solutions were stored at −20 °C.

### 2.2. Instrumentation and chromatographic conditions

Resorufin quantification by HPLC was based on a previously described method [10]. Chromatography was carried out with a pumping system (L-6200A), autosampler (AS 2000), fluorescence detector (L-7480) and D-6000 HPLC Manager software (Merck, Hitachi, Tokyo, Japan). The samples (5 μL) were injected onto a Hypersil ODS column (3 μm, 60 × 4.6 mm, Hewlett–Packard) equipped with a guard column. Resorufin was eluted at a flow rate of 0.8 mL/min of the mobile phase 20 mM phosphate buffer (pH 6.8), methanol and acetonitrile (52:45:3, v/v). Under those chromatographic conditions resorufin was eluted at approximately 1.31 min. The total run time was 7 min. The fluorescence detection was performed at an excitation wavelength of 560 nm and emission wavelength of 586 nm. Additionally, detection of resorufin (EROD activity) in two samples was performed at an excitation wavelength of 540 nm and emission wavelength of 586 nm, and resorufin concentrations in those samples were calculated using a standard curve constructed under the same detection condition.

### 2.3. Standard curve

An eight-point standard curve (0.5, 1.0, 2.5, 5.0, 12.5, 20, 25 and 50 pmol/mL) was prepared by adding known concentrations of resorufin to the mixture of buffer-methanol incubation solution (1:1 v/v).

### 2.4. Porcine hepatic microsome preparation

Pigs used in this study were born and raised at the Swedish University of Agricultural Sciences Funbo-Lövsta xperimental station [15]. Liver samples were collected at slaughter from entire and surgically castrated male pigs, immediately frozen in liquid nitrogen and stored at −80 °C until required for microsome preparations. The microsomal fraction was prepared from the liver homogenate by the Ca-aggregation method as described by Nicolau-Solano *et al*. [16] with slight modifications. Briefly, frozen liver tissue (2.5 mg) was homogenized with ice-cold 10 mM Tris-HCl buffer (5 mL) containing 250 mM sucrose at pH 7.4. The homogenized tissue was centrifuged at 10,000 × g for 10 min at 4 °C. The pellet was discarded and to the supernatant calcium chloride (8 mM) was added, it was well mixed and allowed to stand at 4 °C for 4 min. The supernatants were then centrifuged at 25,000 × g for 30 min at 4 °C to separate the microsomal and cytosolic fractions. The microsomal pellet was resuspended in 50 mM Tris-HCl containing 0.1 mM EDTA and 20% glycerol at pH 7.4. The microsomal protein concentrations were assayed with a commercially available kit (Bio-Rad laboratories Inc., Hercules, CA, USA) according to the manufacturer’s instructions, using bovine serum albumin as a standard. The prepared microsomes were stored at −80 °C until required for assay.

### 2.5. EROD and MROD activity assays

The *O*-dealkylations of ethoxyresorufin and methoxyresorufin in porcine liver were determined using a modification of the method described by Wanwimolruk and Wanwimolruk [11] for Adélie penguin liver. The method was fully validated prior to routine use in our laboratory. Incubation mixtures contained microsomal protein (0.2 mg), phosphate buffer (pH 7.4, 50 mM) and substrate (2 μM; 7-ethoxyresofurin for EROD activity or 7-methoxyresofurin for MROD activity). Reactions were started by the addition of 1 mM NADPH. The reaction mixture, in a final volume of 500 μL, was incubated in a water bath at 37 °C for 5 min. Reactions were terminated with ice-cold 100% methanol (500 μL), followed by centrifugation at 7,500 × g for 5 min. Resorufin concentrations in the supernatants were measured with HPLC the same day as described above. EROD and MROD activities were expressed as pmol of resorufin per milligram protein and minute.

### 2.6. Linearity with incubation time and protein content and stability

A pool of microsomes from one castrated and one entire male pig was used to optimize the incubation conditions. Linear dependency of resorufin formation from 7-ethoxy- or 7-methoxy-resofurin on incubation time was determined using incubation time from 1 to 20 min. Other parameters, such as incubation temperature and protein content (0.2 mg) were kept constant. Linear dependency of resorufin formation on protein content was determined using protein content from 0.1 to 0.6 mg and constant incubation time of 5 min.

### 2.7. Recovery, intra- and inter-assay variations, limit of quantitation and stability

To estimate the accuracy of the method, recovery tests were performed by spiking microsomal incubations (a pool of microsomes from one entire and one castrated male pigs) with known amounts of resorufin (0.5, 10 and 50 pmol/mL). No NADPH was added to the incubations. The recovery was calculated by comparing the response of the incubated resorufin to that of non-incubated resorufin prepared directly in a mixture of incubation buffer-methanol (1:1 v/v). Matrix effect was studied by comparison of resorufin dissolved in a mixture of incubation buffer-methanol (1:1 v/v) and resorufin spiked with microsomal incubations without addition of NADPH. Both sets of samples for matrix effect study were subjected to incubation and centrifugation as described above.

For inter-assay variations the relative standard deviation (RSD) was calculated from five separate measurements on three individual microsomes with different rates of resorufin formation. For intra-assay variations the RSD was calculated on the microsomes repeatable measured within one day.

The limit of quantification for resorufin was defined as the lowest concentration in a sample that can be determined with acceptable precision (20%) under the stated experimental conditions.

The stability was assessed by measuring resorufin in the supernatant (n=8) stored in +4 °C and protected from light for several days and measured at days 0, 1, 2, 3, 5 and 8. A pool of microsomes used for the stability study was previously used for study on linearity with microsomal protein. Resorufin concentrations in those samples varied from 0.9 to 8.1 pmol/mL. Stability of resorufin stored in room temperature was assessed using four samples with resorufin concentrations from 1.5 to 2.6 pmol/mL.

### 2.8. Enzyme kinetic analysis and inhibition constants

The studies were performed by using two pools of microsomes from six entire (pool 1) and six castrated male pigs (pool 2). For the kinetic studies, EROD and MROD activities were determined over the substrate concentration range from 0.0156 to 3.0 μM. For the inhibition study, the substrate range was from 0.25 to 2 µM. The concentrations of inhibitors were chosen based on the results from our pilot study; ANF and ellipticine were added at final concentrations of 0.1, 1.0, 4.0 and 20.0 μM; and furafylline at final concentrations of 10, 20, 50 and 100 μM. The inhibitors were dissolved in DMSO, and the same volume of DMSO was added to the control incubations. The final concentration of DMSO in the incubations was below 0.5%. All inhibitors were added before the addition of the substrate. Furafylline was pre-incubated with the microsomes for 10 min at 37 °C with NADPH before addition of the substrate. Without pre-incubation no inhibition by furafylline was observed. Inhibition is expressed as the percent of the corresponding incubation control.

Michaelis-Menten parameters (K_m_ and V_max_ values) and inhibition constants (K_i_) were determined by a GraphPad Prism version 4.0 for Windows, GraphPad Software (San Diego California, USA). Data were additionally assayed by a SigmaPlot Enzyme Kinetics 1.3 software. Visual analysis of Eadie-Hofstee plots was used to categorise enzyme kinetics as mono- or biphasic, i.e. whether one or more enzymes participate in the reaction. When Eadie-Hofstee plots indicated monophasic kinetics, the following equation was applied to estimate kinetic parameters: V=(V_max_*S)/(K_m_+S); when Eadie-Hofstee plots indicated biphasic kinetics, the following equation was applied: V=(V_max1_*S)/(K_m1_+S)+ (V _max2_*S)/(K_m2_+S).

## 3. Results and Discussion

Accurate measurement of enzyme activities in porcine liver microsomes is important in e.g. endocrinological and biomedical research. Furthermore, it is essential to carefully validate analytical methods for such measurements prior to routine use. The method used in the present study is an adaptation of recently published methods for rapid determination of EROD and MROD activities in the microsomes from various species [10–12]. Here, microsomes from entire and castrated male pigs were used.

### 3.1. Fluorescence detection of resorufin

Resorufin concentrations in two samples measured at different excitation wavelength (540 nm vs 560 nm) and constant emission wavelength (586 nm) did not differ markedly (6.1 vs 5.7 pmol/ml in the sample 1; 7.2 vs 7.2 pmol/mL in the sample 2). In the subsequent experiments, the fluorescence detection was performed at an excitation wavelength of 560 nm and emission wavelength of 586 nm because those conditions resulted in a somewhat higher response for resorufin (data not shown).

### 3.2. Validation of microsomal incubation

Pooled, rather than individual, microsomal preparations from entire and castrated male pigs were used to select suitable incubation time and microsomal protein content for subsequence analyses. Linearity of resorufin formation from 7-ethoxyresorufin was demonstrated up to 7 min of incubation time and 0.3 mg of microsomal protein. Linearity of resorufin formation from 7-methoxyresorufin was up to at least 20 min of incubation time and 0.6 mg of microsomal protein. Microsomal protein of 0.2 mg and incubation time of 5 min were chosen for the subsequent analysis. Resorufin was not detected in the incubations without NADPH, without substrate or without microsomal protein.

The mean recoveries of resorufin ranged between 77.8 – 107.1% (Table 1), which indicates that the method is accurate. The lowest recovery of 77.8% was obtained with lowest concentration of resorufin. In the present study, matrix did not affect the measurements. The differences between blank incubations and incubations of resorufin in the presence of microsomes did not exceed 10%.

**Figure 1.**
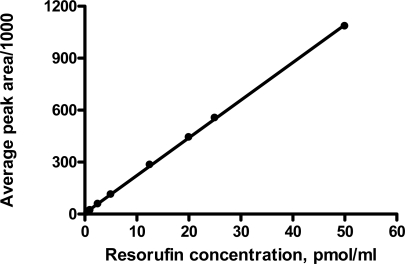
The standard calibration for the quantification of resorufin by HPLC (the linear regression equation for the calibration curve is y = 21.7x + 5.1; coefficient of determination is 0.9998). The fluorescence detection was performed at an excitation wavelength of 560 nm and emission wavelength of 586 nm.

The linear concentration range of the assay was from 0.5 to 50 pmol/mL (Figure 1). Inter-assay variations varied from 3.6 to 15.4% (Table 2). Intra-assay variations did not exceed 12%. The limit of quantitation of resorufin was 0.5 pmol/mL. Taken together, those results demonstrated excellent linearity over the concentration range tested, good accuracy and repeatability, suggesting that the method can be successfully applied to determine EROD and MROD activities in porcine liver microsomes.

Resorufin concentrations in supernatants were stable for at least 5 days if stored at 4 °C under dark conditions. RSD for 8 samples varied from 4.4 to 21.4%; notably the highest RSD (21.4%) at day 5 was obtained when using the sample with the lowest concentration of resorufin (0.9 pmol/ml). At day 8, concentrations of resorufin increased probably due to evaporation of methanol and RSD varied from 7.6 to 26.1%. If stored at room temperature, concentrations of resorufin in the supernatants were stable for at least 1 day (RSD varied from 3.0 to 5.3%).

### 3.3. Kinetic characteristics of EROD and MROD activities

**Figure 2.**
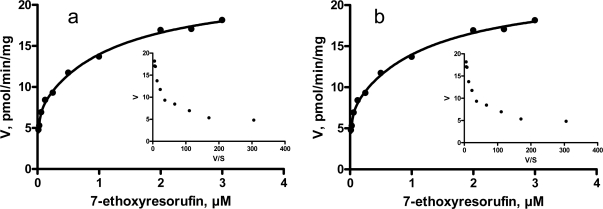
Saturation curve for 7-ethoxyresorufin *O*-deethylation by hepatic microsomes from entire (a) and castrated (b) male pigs. Insets: Eadie-Hofstee transformations of the same data. V – velocity of resorufin formation; S - substrate.

Eadie-Hofstee plots of EROD activities in porcine liver microsomes revealed a biphasic response, indicating that multiple enzymes are responsible for the biotransformation of 7-ethoxyresorufin to resorufin (Figure 2). It has been shown in various studies that EROD activities can be related to both CYP1A1 and CYP1A2 isoenzymes. Messina *et al*. [6] demonstrated that CYP1A2 purified from β-naphthoflavone treated pigs was catalytically active toward 7-ethoxyresorufin and 7-methoxyresorufin. In rat, mouse and penguin liver microsomes, EROD activities followed a biphasic kinetic pattern [10, 11]. In contrary, a single enzyme was responsible for EROD in human, monkey and bovine liver microsomes [10, 12].

As shown in Table 3, K_m_ and V_max_ values for the high-affinity components were fairly constant in entire and castrated male pigs. However, K_m_ and V_max_ for the low-affinity component of EROD and for MROD were somewhat lower in entire male pigs. The reason for this is unclear; it was hypothesized that endogenous steroids may inhibit EROD activities in an uncompetitive manner causing a reduction in K_m_ and V_max_. This, however, should be confirmed. The apparent K_m_ and V_max_ values for low-affinity component of EROD in the present study were fairly similar with those reported for EROD in porcine liver microsomes by Messina *et al*. [6]. Interestingly, the results on kinetic parameters for MROD differed markedly in those two studies. Messina *et al*. [6] demonstrated that K_m_ value (2.2 μM) for MROD in liver microsomes was higher compared to that for EROD (0.64 μM), and compared to the K_m_ values (0.02 and 0.14 μM) found here (Table 3).

**Figure 3.**
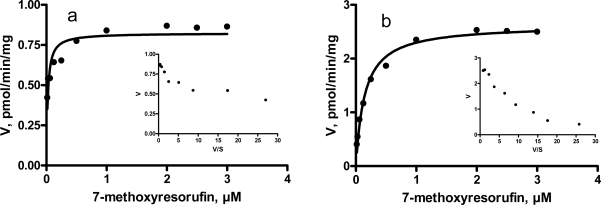
Saturation curve for 7-methoxyresorufin *O*-demethylation by hepatic microsomes from entire (a) and castrated (b) male pigs. Insets: Eadie-Hofstee transformations of the same data. V – velocity of resorufin formation; S - substrate.

Eadie-Hofstee plot of MROD activities was monophasic within the studied concentration range in castrated male pigs, and tended to be monophasic in entire male pigs (Figure 3). Similarly, the kinetics for MROD activities were monophasic in human, monkey, mouse and rat [10].

### 3.4. Inhibition of EROD and MROD activities by α-naphthoflavone (ANF), ellipticine, furafylline

**Figure 4.**
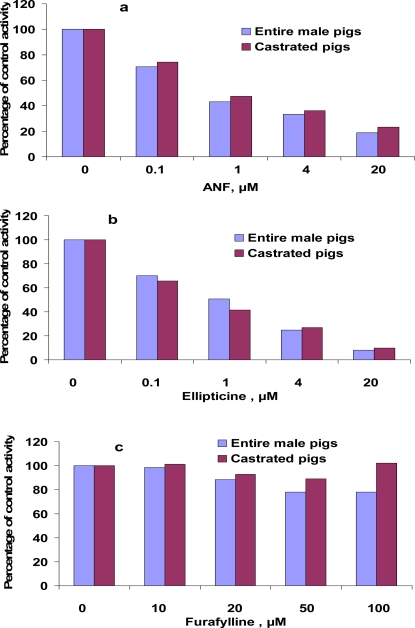
Inhibition of 7-ethoxyresorufin *O*-deethylation (EROD) in hepatic microsomes from castrated and entire male pigs by ANF (a), ellipticine (b) and furafylline (c). Assays were performed as described in the Experimental section. Values are expressed as percentages of activities in control incubations and are the mean of duplicates (differences between the duplicates were below 10%).

**Figure 5.**
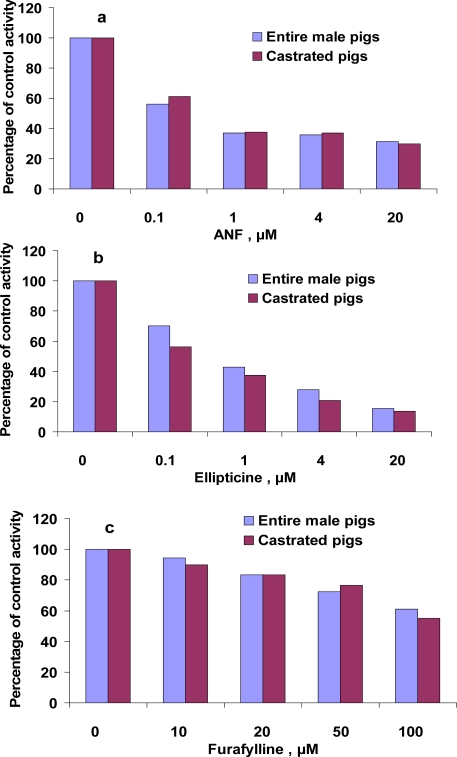
Inhibition of 7-methoxyresorufin *O*-demethylation (MROD) in hepatic microsomes from entire and castrated male pigs by ANF (a), ellipticine (b) and furafylline (c). Assays were performed as described in the Experimental section. Values are expressed as percentages of activities in control incubations and are the mean of duplicates (differences between the duplicates were below 10%).

ANF is generally known as a competitive inhibitor of CYP1A activity in human [17], fish [18] and other species. EROD and MROD activities here were inhibited in a concentration-dependent manner by ANF and ellipticine (Figures 4 and 5). In the presence of 20 μM ANF, EROD and MROD activities decreased to 19–23% (EROD) and to 30% (MROD) of the control values. In the presence of 20 μM ellipticine, which is a specific inhibitor of human CYP1A1 activities [13], EROD and MROD decreased to 8–10% (EROD) and to 14–16% (MROD) of the control values. In some studies on pigs, EROD was used as an indicator of CYP1A1 activities, and MROD as CYP1A2 activities [5]. The fact that MROD activities in the present study were inhibited by a CYP1A1 inhibitor implies the lower selectivity of porcine CYP1A1/2 in catalyzing MROD activities. Alternatively, it might indicate that inhibitors, considered to be specific for human CYP1A isoforms, would not necessary inhibit the corresponding isoform in pigs. This suggestion is in line with observation of monophasic pattern of Eadie-Hofstee plots of MROD. Additionally, a monophasic Eadie-Hofstee plot does not necessarily indicate the involvement of a single enzyme; involvement of several enzymes with similar K_m_ values would also result in a monophasic Eadie-Hofstee plot.

Messina *et al*. [6] showed an inhibition of EROD activities by ANF and ellipticine using pig and human recombinant CYP1A2. It was demonstrated that the expression of protein CYP1A2 was positively correlated with MROD, but not EROD activities [6]. Furafylline, a specific inhibitor of human CYP1A2, inhibited MROD activities (Figure 5), but did not affect EROD activities (Table 4). This inhibition of MROD, however, was weaker compared with inhibition by ANF and ellipticine. As much as 100 μM furafylline was needed to reduce MROD activities to 55–61% of the control values. It should be emphasized that specificity of furafylline as inhibitor of human CYP1A2 was investigated using activity of phenacetin *O*-deethylase as an indicator of CYP1A2 activity [19], whereas here we used 7-methoxyresorufin *O*-demethylation.

Based on the lack of inhibition of EROD activities by furafylline, it can be suggested that EROD is not a marker for CYP1A2 in porcine liver. However, the biphasic pattern of Eadie-Hofstee plots of EROD reflects the involvement of multiple enzymes, suggesting that 7-ethoxyresorufin is not a specific substrate for CYP1A1 activity in pigs. Thus, final conclusion must await isolation and characterization of porcine CYP1A1. The question whether the second EROD isozyme in porcine microsomes is a CYP1A2, remains to be answered.

No inhibition of MROD activity by furafylline was observed when no pre-incubation of microsomes and furafylline with NADPH was performed (data not shown). This observation is consistent with results from previous studies on the mechanism of furafylline inhibition of MROD. Ueng *et al*. [20] demonstrated that pre-incubation of mouse microsomes and furafylline with an NADPH-generating system resulted in strong inhibition of MROD activity. In contrast, Schultz *et al*. [21] found no differences when furafylline was incubated with microsomes from marmosets and NADPH before addition of substrate, or when furafylline and substrate were incubated together before the addition of NADPH. Inhibition constants (K_i_) of the inhibitors on EROD and MROD are shown on Table 4. It is unclear whether the observed differences in K_i_ between two groups of microsomes are due to the presence of endogenous steroids in the liver from entire male pigs or some other unknown factors. The gender-related difference in EROD and MROD activities in porcine liver microsomes was recently established [5]. Further studies on the effect of high levels of testicular steroids on EROD and MROD activities are in progress in our laboratory.

## 4. Conclusions

The results from the current study demonstrated that an HPLC-based method previously developed to measure EROD activities in several species can be applied to measure EROD and MROD activities in porcine liver microsomes with high accuracy and repeatability. EROD activities in porcine liver microsomes were inhibited by ANF and a specific inhibitor of human CYP1A1 ellipticine, but not by specific human CYP1A2 inhibitor furafylline. MROD activities were strongly inhibited by ellipticine and to a much lesser extent by furafylline. It might indicate that inhibitors, considered to be specific for human CYP1A isoforms, would not necessary inhibit the corresponding isoform in pigs. This stresses the importance in evaluation of the potential substrates and inhibitors to characterize individual CYP enzymes in different animal species. Further studies are needed to evaluate substrate specificities of porcine CYP1A1 and CYP1A2 as well as extents to which CYP1A1 and CYP1A2 contribute to EROD and MROD activities.

## Figures and Tables

**Table 1.** Assessment of recovery and matrix effect on resorufin measurements

Added concentration of resorufin, pmol/mL	Concentration of resorufin measured in blank incubations, pmol/mL (recovered, %)	Concentration of resorufin measured in microsomal incubations, pmol/mL (recovered, %)
0.5	0.45 (90.4)	0.39 (77.8)
10	10.3 (103.5)	10.7 (107.1)
50	49.2 (98.3)	47.7 (95.3)

**Table 2.** Inter-assay variations in resorufin formation from 7-ethoxyresorufin and 7-methoxyresorufin.

Sample	EROD			MROD		

	1	2	3	1	2	3
Mean, pmol/min/mg	14.4	42.4	55.6	5.1	7.8	14.5
SD	1.3	1.5	8.5	0.57	0.88	0.69
RSD,%	9.3	3.6	15.4	11.1	6.1	8.8

EROD: ethoxyresorufin *O*-deethylation; MROD: methoxyresorufin *O*-demethylation; SD: standard deviation; RSD: relative standard deviation

**Table 3.** Kinetic parameters of resorufin formation from 7-ethoxyresorufin and 7-methoxy-resorufin in the microsomes from entire male and castrated pigs.

		Kinetic parameter	Incubations	
			Entire male pigs	Castrated male pigs
EROD	High-affinity component	K_m_, μM (CI)	0.01 (0.005 – 0.022)	0.01 (0.001 – 0.020)
		V_max_, pmol/min/mg (CI)	5.9 (3.1 – 8.6)	7.3 (5.5 – 9.0)
	Low-affinity component	K_m_, μM (CI)	0.54 (0.10 – 0.99)	1.25 (0.37 – 2.14)
		V_max_, pmol/min/mg (CI)	11.7 (9.6 – 13.8)	15.2 (12.9 – 17.6)
MROD		K_m_, μM (CI)	0.02 (0.75 – 0.89)	0.14 (0.11 – 0.18)
		V_max_, pmol/min/mg (CI)	0.8 (0.7 – 0.9)	2.6 (2.5 – 2.8)

EROD: ethoxyresorufin O-deethylation; MROD: methoxyresorufin O-demethylation; CI: 95% confidence interval. The K_m_ and V_max_ values and CI were calculated using nonlinear regression analysis with GraphPad Prism program 4.0 kinetic software.

**Table 4.** Inhibition constants of ANF, ellipticine and furafylline for EROD and MROD in the microsomes from castrated and entire male pigs.

Inhibitor	Ki, μM			
	EROD		MROD	

	Entire male pigs	Castrated pigs	Entire male pigs	Castrated pigs
ANF	0.031	0.096	0.014	0.018
Ellipticine	0.017	0.021	0.024	0.019
Furafylline	no*	no*	102.7	37.6

ANF: α-naphthoflavone; EROD: ethoxyresorufin O-deethylation; MROD: methoxyresorufin *O*-demethylation. The inhibitior constants K_i_ were calculated using nonlinear regression analysis with GraphPad Prism program 4.0 kinetic software.

* No inhibition was observed within the range of substrate concentrations used.
